# Effects of Hst3p inhibition in *Candida albicans*: a genome-wide H3K56 acetylation analysis

**DOI:** 10.3389/fcimb.2022.1031814

**Published:** 2022-10-27

**Authors:** Marisa Conte, Daniela Eletto, Martina Pannetta, Anna M. Petrone, Maria C. Monti, Chiara Cassiano, Giorgio Giurato, Francesca Rizzo, Peter Tessarz, Antonello Petrella, Alessandra Tosco, Amalia Porta

**Affiliations:** ^1^ Department of Pharmacy, University of Salerno, Fisciano, Salerno, Italy; ^2^ Ph.D. Program in Drug Discovery and Development, University of Salerno, Fisciano, Salerno, Italy; ^3^ Department of Pharmacy, University of Naples ‘Federico II’, Naples, Italy; ^4^ Laboratory of Molecular Medicine and Genomics, Department of Medicine, Surgery and Dentistry “Scuola Medica Salernitana”, University of Salerno, Baronissi, Salerno, Italy; ^5^ Max Planck Research Group “Chromatin and Ageing”, Max Planck Institute for Biology of Ageing, Cologne, Germany; ^6^ Cologne Excellence Cluster on Stress Responses in Ageing-Associated Diseases (CECAD), University of Cologne, Cologne, Germany

**Keywords:** *Candida*, sirtuin, Hst3p deacetylase, H3K56 acetylation, ChIP-seq, antifungals

## Abstract

*Candida* spp. represent the third most frequent worldwide cause of infection in Intensive Care Units with a mortality rate of almost 40%. The classes of antifungals currently available include azoles, polyenes, echinocandins, pyrimidine derivatives, and allylamines. However, the therapeutical options for the treatment of candidiasis are drastically reduced by the increasing antifungal resistance. The growing need for a more targeted antifungal therapy is limited by the concern of finding molecules that specifically recognize the microbial cell without damaging the host. Epigenetic writers and erasers have emerged as promising targets in different contexts, including the treatment of fungal infections. In *C. albicans*, Hst3p, a sirtuin that deacetylates H3K56ac, represents an attractive antifungal target as it is essential for the fungus viability and virulence. Although the relevance of such epigenetic regulator is documented for the development of new antifungal therapies, the molecular mechanism behind Hst3p-mediated epigenetic regulation remains unrevealed. Here, we provide the first genome-wide profiling of H3K56ac in *C. albicans* resulting in H3K56ac enriched regions associated with *Candida* sp. pathogenicity. Upon Hst3p inhibition, 447 regions gain H3K56ac. Importantly, these genomic areas contain genes encoding for adhesin proteins, degradative enzymes, and white-opaque switching. Moreover, our RNA-seq analysis revealed 1330 upregulated and 1081 downregulated transcripts upon Hst3p inhibition, and among them, we identified 87 genes whose transcriptional increase well correlates with the enrichment of H3K56 acetylation on their promoters, including some well-known regulators of phenotypic switching and virulence. Based on our evidence, Hst3p is an appealing target for the development of new potential antifungal drugs.

## Introduction

The polymorphic fungus *Candida albicans* is the major human opportunistic fungal pathogen (Lohse et al., 2018); its medical importance makes it a suitable experimental model for studying fungal pathologies and the underlying biology of dimorphic fungi (Kabir et al., 2012). The pathogenic cues for *C. albicans* are mostly environmental shifts such as a compromised immune system, a change of microbial flora following antibiotic treatment, or a medical device implant and organ transplantation (Lamoth et al., 2018). The ability of *C. albicans* to survive and proliferate in hostile environments (Mayer et al., 2013), combined with the dynamic morphological transition from yeast to filament form, is the most noticeable determinant for virulence rather than its static morphology, yeast or hypha, itself (Sudbery et al., 2004; Kadosh, 2016). Therefore, transcriptional regulation behind this dynamic morphology is directly linked to the control of *C. albicans* virulence. As in all eukaryotes, both transcriptional repression and activation are directly influenced by chromatin structures, whose dynamic change depends mainly on posttranslational histone modifications (Fallah et al., 2021). Indeed, many studies revealed that the epigenetic regulation by chromatin structure modifiers, along with well-known transcription factors in the signaling pathways, could be linked to the morphological phenotype transition (Klar et al., 2001; Simonetti et al., 2007; Zacchi et al., 2010; Rai et al., 2018). Specifically, the yeast to hyphae transition, the white-opaque switching, the ability to develop biofilm, and the adaptation to drug pressure are interesting and essential pathogenic mechanisms of this fungus, and posttranslational histone modifications play a prominent role in their regulation (Hnisz et al., 2009; Li et al., 2015; Kim et al., 2015; Garnaud et al., 2016; Qasim et al., 2021).

Among various chromatin modifications, histone acetylation-deacetylation plays a leading role since it regulates pathogenic processes and promotes *C. albicans* virulence (Simonetti et al., 2007; Zacchi et al., 2010; Garnaud et al., 2016).

In *C. albicans*, histone deacetylases (HDAC) have been divided into three groups: Class I and Class II HDACs, which are zinc-dependent, and Class III HDACs, namely the sirtuin family, which are NAD^+^ dependent (Su et al., 2020). Recent studies showed that sirtuins might be involved in sensing environmental changes triggering morphological transition (Zhao and Rusche, 2021). In *C. albicans*, there are five sirtuin-coding genes (*SIR2*, *HST1*, *HST2*, *HST3*, and orf19.2963) with similarities to *Homo sapiens SIRT5*. Sir2p and Hst1p are paralogs resulting from an ancient gene duplication, whereas Hst2p is evolutionarily more divergent (Rupert et al., 2016). However, they have distinct roles and different localization in *C. albicans* cells, as Sir2p localizes in the nucleolus, Hst1p in the nucleus, and Hst2p in the cytoplasm (Zhao and Rusche, 2021). In particular, Hst1p, a component of the Set3 complex (Set3C), is thought likely to repress yeast-to-filament transition by attenuating the cAMP/PKA signaling pathway while promoting the white-to-opaque switching (Su et al., 2020). On the other hand, a decrease in the expression of hypha-specific genes *HWP1, ALS3*, and *ECE1* was observed in the sir2Δ/Δ mutant (Zhao and Rusche, 2021).

In *C. albicans*, the NAD^+^-dependent histone deacetylase (sirtuin) Hst3p is responsible for removing the acetyl group of the Lys 56 of the H3 histone, which is particularly abundant in yeasts; it marks newly synthesized histones, facilitating their deposition onto chromatin and chromatin segments with high nucleosome turnover. This posttranslational modification is significant in yeasts because it regulates DNA damage response and contributes to fungal genome integrity (Celic et al., 2006; Wurtele et al., 2010). In *C. albicans*, H3K56 acetylation/deacetylation balance is regulated by the acetyltransferase (HAT) Rtt109p and Hst3p deacetylase, encoded by *RTT109* and *HST3* genes, respectively (Wurtele et al., 2010).

Previous studies highlighted that *HST3* deletion is lethal for *C. albicans* (Wurtele et al., 2010), suggesting that this is an essential gene in this fungus. In addition, *HST3* conditional mutants displayed attenuated virulence in mice models; moreover, a reduced copy number of *HST3*, or inhibition of Hst3p by nicotinamide (NAM) treatment, promoted white-to-opaque switching with a consequent increased opaque phenotype (Wurtele et al., 2010; Stevenson and Liu, 2011; Stevenson and Liu, 2013; Guan and Liu, 2015).

Like antibiotic-resistant bacteria, the occurrence of fungal strains resistant to the main antifungal drugs (including polyenes, azoles, echinocandins, and 5-Flucytosine) is becoming a serious threat to worldwide public health. Moreover, the available therapeutic molecules lack specificity and selectivity with consequent multiple side effects. Given the central role of histone acetylation/deacetylation balance in *C. albicans* growth and virulence, HATs and HDACs represent a promising target for developing new antifungal agents.

Fungal enzymes that regulate H3K56ac levels diverge significantly from their human counterparts; indeed, fungal Hst family members share sequence motifs absent in human sirtuins (Wurtele et al., 2010). In addition, Hst3p has high substrate specificity, unlike the sirtuins Sirt1 and Sirt2 involved in H3K56 deacetylation in human cells (Celic et al., 2006). Based on this evidence, Hst3p represents a unique and interesting target for the development of new antifungals.

Despite ongoing efforts over recent years, the roles played by Hst3p and H3K56 acetylation dynamics are only partially understood. Therefore, in this study, using the non-specific sirtuin inhibitor NAM, a product of NAD^+^-dependent deacetylation reaction, we evaluated the effect of Hst3p inhibition on the acetylation levels of its substrate H3K56 during yeast growth as well as on morphology and transcription profile of *C. albicans*. Moreover, by Chromatin immunoprecipitation followed by sequencing (ChIP-seq) analysis, we provide, for the first time, a genome-wide map of H3K56ac and identify some virulence-related genes whose transcription is directly or indirectly regulated by Hst3p.

## Materials and methods

### Chemicals

NAM was purchased by Sigma-Aldrich (Milan, Italy). For all the experiments, 2 M NAM in ultra-pure distilled water was used as stock and added to cultures to obtain the required concentrations. Sirtinol, SirReal2, and Inauhzin, purchased from Selleckchem (Planegg, Germany), were dissolved in dimethylsulfoxide (DMSO) (Sigma-Aldrich, Milan, Italy).

### Fungal strain and growth conditions

*C. albicans* wild-type strain SC5314 (ATCC-MYA-2876) was routinely cultured on YPD (1% Yeast extract, 2% Peptone, 2% Dextrose) agar plates and propagated in liquid YPD medium overnight at 25°C at 200 rpm. Depending on experimental conditions, *C. albicans* was grown in synthetic YNB medium (0.17% Difco Yeast Nitrogen Base, without amino acids and ammonium sulfate), supplemented with 2% glucose and 0.5% ammonia sulfate; M199 medium containing Earle’s salts and glutamine (Sigma-Aldrich, Milan, Italy), buffered at pH 7.5 with 25 mM HEPES; 10% FBS (fetal bovine serum) (Euroclone, Pero, Italy); RPMI 1640 medium with L-Glutamine (Euroclone, Pero, Italy), Spider medium (2% mannitol, 2% nutrient broth, 0.4% K_2_HPO_4_, pH adjusted to 7.2 with NaOH). Each medium was supplemented with 10 or 25 mM NAM depending on the experimental needs. Solid media were prepared by adding 2% agar to the liquid broth before autoclaving. The optical density (OD) of each culture was measured at a wavelength of 600 nm (OD_600_). Three independent biological replicates were performed.

### NAM, Inauhzin, Sirtinol, and SirReal2 treatment

A budding yeast culture was diluted in 6-well plates to a density of 10^5^ cell/ml in 2 mL YPD with 10 mM NAM (CaNAM), 50 µM Inauhzin, 10 µM Sirtinol or 50 µM SirReal2. *Candida* cells treated with only vehicle (DMSO) were used as control (CTRL). Plates were incubated at 30°C for 28 hours, and cell differentiation was followed by Time Lapse imaging (Leica DMI6000 T, Buccinasco, Italy).

A total of 5 x 10^8^ cells were harvested by centrifugation (4,700*g*, 10 min at 4°C), washed with distilled water, and then stored at -80°C for subsequent histone extraction. Three independent biological replicates were performed.

### *Candida* growth curve

Over-weekend yeast cell culture was diluted to 10^7^ cells/mL in 50 mL YPD medium and allowed to grow at 25°C overnight. Subsequently, the budding yeast culture was used to inoculate 700 mL YPD (with and without 10 mM NAM) at a cell density of 2x10^6^/mL. Treated (CaNAM) and untreated yeast cells (CTRL) were cultured for 50 hours with orbital shaking (200 rpm) at 25°C, and growth was followed by measuring the OD_600_. At selected time points (0, 2, 4, 6, 8, 10, and 28 h), a pellet of 5 x 10^8^ cells was collected by centrifugation (4700*g*, 10 min at 4°C), washed with distilled water, and then stored at -80°C for subsequent histone extraction.

### H3K56 acetylation levels during yeast, germ tube, and hyphae growth

*C. albicans* grown overnight in YNB at 25°C were collected by centrifugation (2,000*g*, 10 min at 25°C), washed with 0.15 M NaCl, resuspended in 0.15 M NaCl, and incubated at 25°C for 24 h to induce starvation. Subsequently, 10^6^ yeasts/mL were inoculated in RPMI 1640 for 6 hours at 25, 30, or 37°C so that the cell morphology was regulated only by the growth temperature, in particular, obtaining yeast cells at 25°C, germ tubes at 30°C and true hyphae at 37°C.

Cells were harvested by centrifugation (4700*g*, 10 min at 4°C), the pellets were washed with distilled water, and then stored at -80°C for subsequent histone extraction. Three independent biological replicates were performed.

### *Candida* histones extraction

*C. albicans* pellets were first resuspended in 10 mM EDTA, 10 mM Tris-HCl pH 7.4, 5 mM sodium butyrate, 5 mM NAM, 2.5% 2-Mercaptoethanol and 10% glycerol. The cellular suspension was ground to a fine powder with a mortar and pestle in liquid nitrogen. To enhance the lysis efficiency, the powder was resuspended in 10 mM EDTA, 10 mM Tris-HCl pH 7.4, 5 mM sodium butyrate, 5 mM NAM, 2.5% 2-Mercaptoethanol, 1% SDS, and 2% Triton-X 100 and acid-washed glass beads were added. Two or more cycles of freeze and vortex were performed. The suspensions were centrifuged at 12,000*g* for 15 min at 4°C, and the supernatants were collected.

Acid soluble proteins were extracted from the total lysates by adding 0.4 N H_2_SO_4_ followed by incubation at 4°C for 3 h, under gentle inversion. Histones were acid-precipitated with 25% TCA (trichloroacetic acid, Sigma-Aldrich, Milan, Italy) overnight at 4°C. After two washing steps with ice-cold acetone, precipitated proteins were resuspended in Milli-Q H_2_O. Histones were resolved on SDS-PAGE (15% polyacrylamide gel), and the gel was stained with Coomassie G-250 Brilliant Blue (Sigma-Aldrich, Milan, Italy) and destained in water.

For Western blotting, 0.5 μg of each histones sample were resolved on SDS-PAGE and transferred to a nitrocellulose membrane using the Trans-Blot Turbo Transfer System (Bio-Rad, Segrate, Italy).

The following primary antibodies were used for detection: H3 (Abcam, ab1791, Cambridge, UK) and H3K56ac (Active Motif, Waterloo, Belgium). Band densities were visualized by LAS 4000 (GE Healthcare, Life Sciences) digital imaging system and quantified using ImageJ analysis software.

### Analysis of H3K56ac by Nano LC-MS/MS

Histones H3 gel bands were cut from the Coomassie-stained gel and subjected to trypsin *in situ* digestion, as described by Shevchenko et al. (Shevchenko et al., 2006). Extracted peptides were dissolved in 10% formic acid before nano-ESI-LC-MS/MS analysis. Peptides were separated by a nano Acquity LC system (Waters Corp. Manchester, UK) equipped with a BEH C-18 1,7 µm, 75 µm x 250 mm (Waters Corp. Manchester, U.K) column connected to LTQ-Orbitrap hybrid mass spectrometer (Thermo Scientific). 5 μL of each sample were loaded onto the column and separated at a flow rate of 280 nL/min in a 15% - 40% buffer B linear gradient (Buffer A: 95% H_2_O, 5% ACN, 0.1% AA; Buffer B: 95% ACN, 5% H_2_O, 0.1% AA) in 55 minutes. Nano-ESI-LC-MS/MS analyses of H3 tryptic peptides were performed using Selected Reaction Monitoring (SRM) method on LTQ-Orbitrap mass spectrometer.

The amount of acetylated peptide of interest (FQK(ac)STELLIR) was quantified by monitoring its bi-charged ion at m/z 638.82 and its fragmentation which produced two ions (831.54 and 1001.61). H3 peptide YKPGTVALR, an unmodified peptide, was used to normalize H3 quantities in each gel band, monitoring its bi-charged ion at m/z 502.86 and the fragmentation that produced two ions at m/z 616.38 and 713.50.

### Morphological analysis on solid agar media

To evaluate morphologic changes induced by NAM treatment, *C. albicans* yeasts grown in YNB at 25°C were diluted to a cell density of 2 x 10^7^ cells/mL, and 5 μL of this dilution were spotted onto solid media containing or not 25 mM NAM. In particular, YPD agar plates were incubated at 25°C, whereas 10% FBS, M199, and Spider medium were incubated at 37°C for hyphal growth induction. After 72 h of incubation, colony morphology was examined by microscopy at 10x magnification using an AMG Evos Imaging System (Thermo Fischer Scientific, Monza, Italy).

### Chromatin immunoprecipitation

Budding yeast cell culture was diluted at a cell density of 10^5^ cells/mL, distributed in Petri dishes of 90 mm diameter (10 mL each), and incubated at 25°C for 28 h with or without 10 mM NAM. Afterward, *C. albicans* cultures were cross-linked with 1% formaldehyde for 15 minutes at room temperature with gentle shaking. The reaction was quenched by adding 125 mM glycine and incubating for 5 min at room temperature under gentle shaking. Chromatin immunoprecipitation (ChIP) was performed as previously described (Mawer et al., 2021), except for cell lysis that was carried out by using a cryogenic freezer mill (SPEX SamplePrep 6970EFM Freezer/Mill, München, Germany). ChIP-seq libraries were generated from two independent biological replicates of H3K56ac and input following a previously published protocol (Ford et al., 2014) and sequenced on Illumina NextSeq 500 using 2 × 75 bp reads. Two independent biological replicates were performed for either control cells (CTRL) or *C. albicans* treated with 10 mM NAM (CaNAM).

### Total RNA extraction

As described above, *C. albicans* cultures were grown with or without 10 mM NAM in Petri dishes of 90 mm diameter and incubated at 25°C. Three independent biological replicates were performed for either CTRL or CaNAM cells. After 28 h of incubation, a total of 10^9^ yeast cells were harvested (8,000g, 10 min at 4°C) and washed with diethylpyrocarbonate (DEPC)-treated water. RNA isolation was performed as described by Vennapusa et al., 2020 with some modifications. Briefly, yeast cells were resuspended in 600 mL of RNA extraction buffer (100 mM Tris- HCl (pH: 8), 25 mM EDTA, NaCl 2.5 M, 2.5% β-Mercaptoethanol, 2% SDS) and disrupted mechanically with BeadBug microtube homogenizer (Benchmark Scientific, Sigma-Aldrich, Milan, Italy) by using acid-washed glass beads.

Total RNA was isolated using an equal volume of Acid-Phenol/Chloroform, pH 4.5 with IAA, 25:24:1 (Sigma-Aldrich, Milan, Italy). Following centrifugation, the RNA was precipitated from the aqueous phase with 100% ethanol, washed twice with 75% ethanol, and dissolved in DEPC-water.

RNA quantification was carried out with the instrument Nanodrop 200 Thermo Fisher Scientific (Monza, Italy).

### RNA sequencing

For RNA sequencing, RNA quality was assessed with TapeStation (Agilent, Cernusco sul Naviglio, Italy), and only RNA with RIN > 8 was used for library production. According to the manufacturer instructions, indexed libraries were prepared from 1 µg of purified RNA using TruSeq Stranded Total RNA Sample Prep Kit (Illumina Inc., Berlin, Germany). Libraries were pooled and sequenced (paired-end, 2 x 100 bp) on NextSeq 550 platform (Illumina Inc., Berlin, Germany). A Ribo-Zero Gold rRNA Removal Kit specific for yeasts was used (Illumina Inc., Berlin, Germany) to remove rRNA. Libraries were pooled and sequenced (paired-end, 2 x 75 cycles) on NextSeq 550 platform (Illumina Inc., Berlin, Germany). Three independent biological replicates were performed.

### Bioinformatic analysis

For RNA sequencing, the raw sequence files generated (.fastq files) underwent quality control analysis using the FastQC tool (http://www.bioinformatics.babraham.ac.uk/projects/fastqc), and the quality-checked paired-end reads were then aligned to the reference *Candida albicans* SC5314 genome (assembly GCA_000182965.3) using STAR (version 2.5.2a) (Dobin et al., 2013), with standard parameters. The FeatureCount algorithm (Liao et al., 2014) was used to quantify each transcript using the reads mapped to the genome. A given gene was considered expressed when detected by at least 10 total reads in the 3 replicates. Data normalization and differentially expressed transcripts were identified using DESeq2 (Love et al., 2014) with standard parameters; differential expression was reported as fold change. A gene with FDR ≤ 0.05 (False Discovery Rate) and with a value of Fold Change ≤ -1.5 (for down-regulated genes) or Fold Change ≥ 1.5 (for up-regulated genes) was considered significantly differentially expressed.

For ChIP-sequencing, the analysis was performed using the Galaxy tool (v 22.05) (https://usegalaxy.eu/) (Galaxy Community, 2022). Briefly, after the FastQC quality check, the paired-end reads were aligned to the reference *Candida albicans* SC5314 genome (assembly GCA_000182965.3) using Bowtie 2 (Galaxy Version 2.4.4), and the generated BAM files were filtered with Filter BAM (-q=20) (Galaxy Version SAMTOOLS: 1.8). Mapped reads were indexed and merged using samtools MergeSamFiles (Galaxy Version 2.18.2.1) and converted to bigwig files using deepTools bamCoverage (Galaxy Version 3.5.1.0.0) with a bin size of 10 and normalization to genomic content. Peak calling was performed with MACS2 callpeak (Galaxy Version 2.2.7) using standard parameters for board regions and normalized to the effective genome size. Peak annotation was carried out using ChIPseeker (Galaxy Version 1.28.3).

In both ChIP-seq and RNA-seq analyses, PCR duplicates were excluded. Network analysis was performed using ClueGo and CluePedia Cytoscape plugins (version 3.9.1) (Bindea et al., 2009). Venn diagrams were designed by InteractiVenn (Heberle et al., 2015).

### Statistical analysis

Data are from at least three independent experiments, and results are expressed as means ± SD. Data were analyzed with GraphPad Prism 7 (GraphPad Software). Two-tailed Student’s t-test (2-group comparisons) or two-way ANOVA (>2-group comparisons) were performed as appropriate. P values < 0.05 were considered significant.

## Results

### NAM treatment causes an accumulation of acetylated H3K56

**Figure 1:**
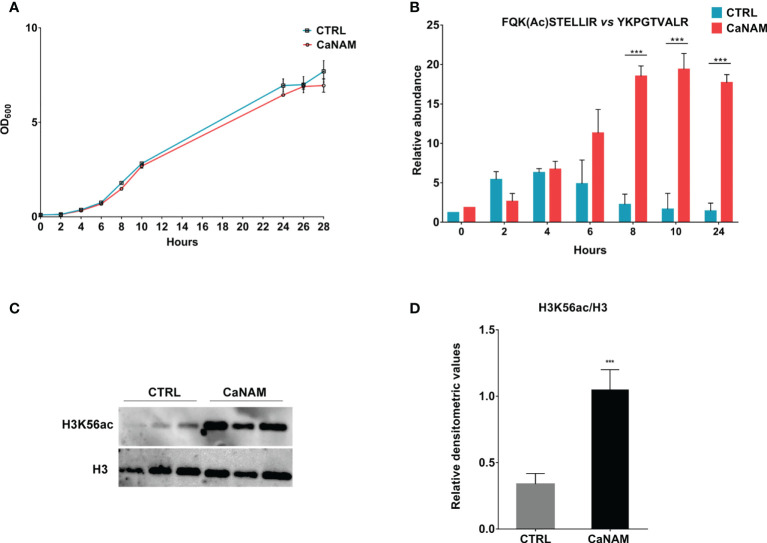
H3K56 acetylation levels during *C. albicans* growth. **(A)** Comparison of the growth curve of *Candida* yeast control versus treated with 10 mM NAM. **(B)** Quantitation of H3K56ac levels during *C. albicans* growth, with and without 10 mM NAM by Nano LC-MS/MS. **(C)** Representative Western Blot showing H3K56ac accumulation after 28h of treatment with 10 mM NAM. **(D)** Densitometric analysis of H3K56ac normalized to H3 from three independent experiments as in **(C)**. Values are mean ± standard error of three independent experiments ***p<0.001 (t-test).

To determine the minimum concentration of NAM able to induce an accumulation of H3K56ac without affecting *Candida* growth within 28 h, different concentrations of NAM (5-100 mM) were assayed (data not shown). As shown in **Figure 1A**, 10 mM NAM did not significantly affect fungus duplication and, more importantly, caused a robust accumulation of H3K56 acetylation levels (**Figures 1B-D**). During *C. albicans* growth, nuclear protein fractions were prepared from selected time points (0, 2, 4, 6, 8, 10, and 28 h) from both treated and untreated cells to determine the acetylation levels of H3K56 by nanoscale Liquid Chromatography coupled to tandem Mass Spectrometry (nano-ESI-LC-MS/MS). The quantification of FQK(Ac)STELLIR, the histone tryptic peptide including the acetylated lysine, was performed by normalizing its chromatographic peak area *versus* YKPGTVALR, another tryptic peptide of H3 histone which does not display post-translational modifications. Nano-ESI-LC-MS/MS analysis revealed a maximum peak of H3K56 acetylation after 4 h-inoculation and a decrease over time in untreated control cells. On the contrary, the acetylation levels of H3K56 upon NAM treatment increased and accumulated during growth, reaching a plateau up to 24 h (**Figure 1B**). These results describe for the first time how H3K56 is acetylated during *C. albicans* yeast growth and confirm the inhibitory effect of NAM on the fungal sirtuin Hst3p. The NAM-depending accumulation of H3K56ac was also confirmed by Western blotting of histones isolated from *Candida* after 28 h-incubation (**Figures 1C, D**).

### Hst3p inhibition alters *C. albicans* morphology

As a dimorphic fungus, *C. albicans* can reversibly switch from the yeast morphology to elongated hyphal forms, responding to environmental stimuli in a finely regulated process characterized by extensive changes in gene expression profile.

Given the central role of the chromatin landscape in transcription activation/repression in eukaryotes and considering that H3K56 acetylation is the most abundant post-translational modification in *C. albicans*, we wondered whether such histone modification might be involved in the regulation of hypha-specific genes (HSG). To this end, we analyzed the colony morphology of *C. albicans* resulting from inhibition of Hst3p.

**Figure 2:**
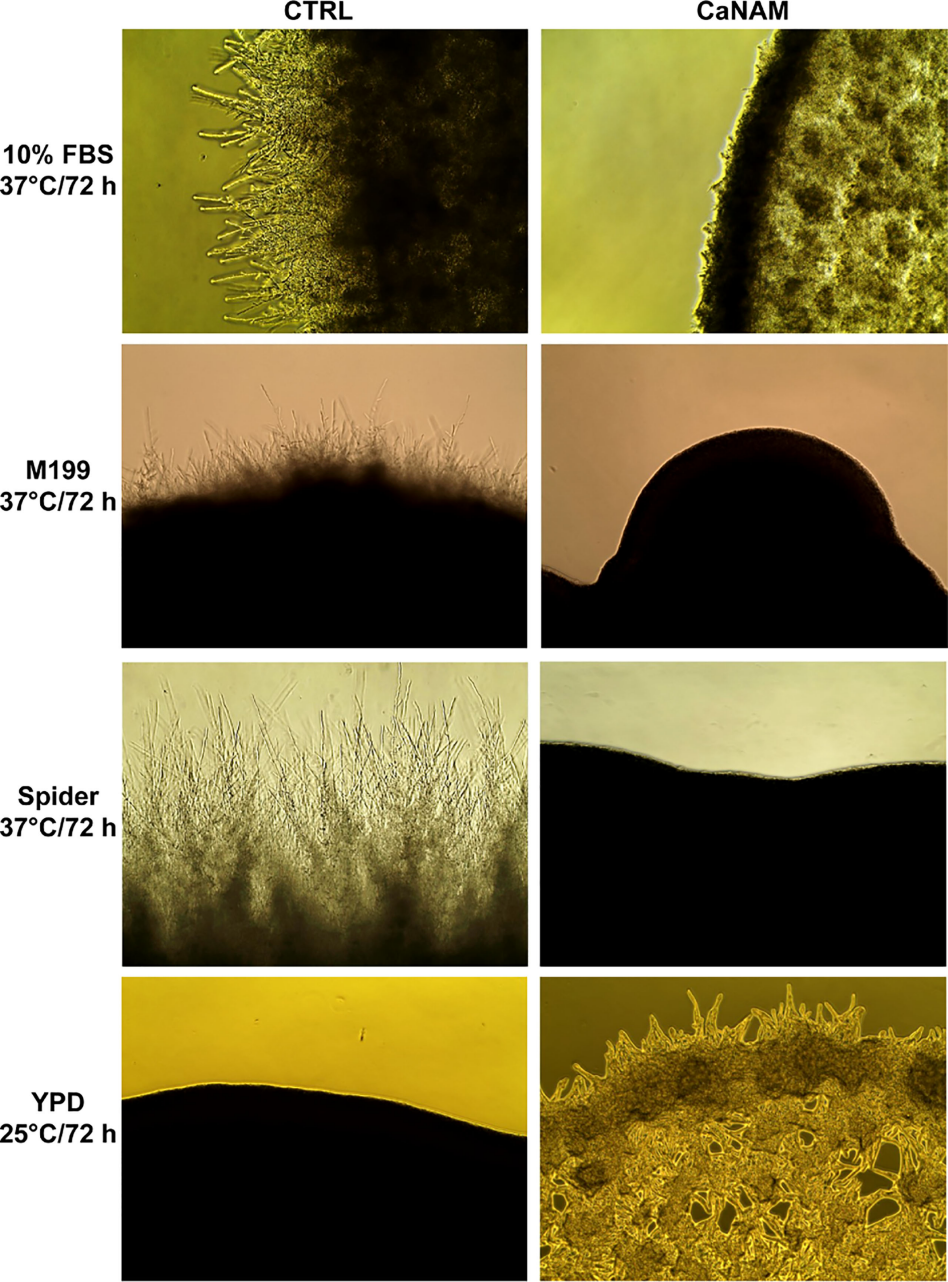
Colony morphologies of *C. albicans* grown on different media with or without 25 mM NAM. *C. albicans* was grown in the indicated media, w/wo NAM, for 72 h, and the morphology was examined by optical microscopy at 10X magnification. The addition of 25 mM NAM inhibits the filamentation in hyphae-inducing conditions (10% FBS, M199 or Spider Medium; 37°C), whereas in the yeast-promoting condition (YPD; 25°C), the treatment with NAM triggers the formation of abnormal and enlarged filamentous structures called V-shaped hyphae.

The filamentation was induced with 10% serum, M199, or Spider medium at 37°C, in the presence or absence of 25 mM NAM. As reported in **Figure 2**, 25 mM NAM led to a significant alteration in *C. albicans* morphology with a robust inhibition of filamentation and formation of hyphal crown around the macro-colonies on solid media. In addition, according to Wurtele and colleagues (Wurtele et al., 2010), we observed the formation of abnormal and enlarged filamentous structures, with a particular conformation called V-shaped hyphae, when Hst3p was inhibited under yeast-promoting conditions (YPD at 25°C) (**Figure 2**).

Such morphological alteration supports the hypothesis that H3K56ac might be involved in the yeast-to-hyphae transition. However, Western blotting analysis of histones isolated respectively from yeast, germ tube, and hyphal shape revealed that the overall acetylation levels of H3K56 are comparable among the three cellular forms (**Supplementary Figure S1**).

### Sirtinol, SirReal2, and Inauzhin do not inhibit Hst3p

**Figure 3:**
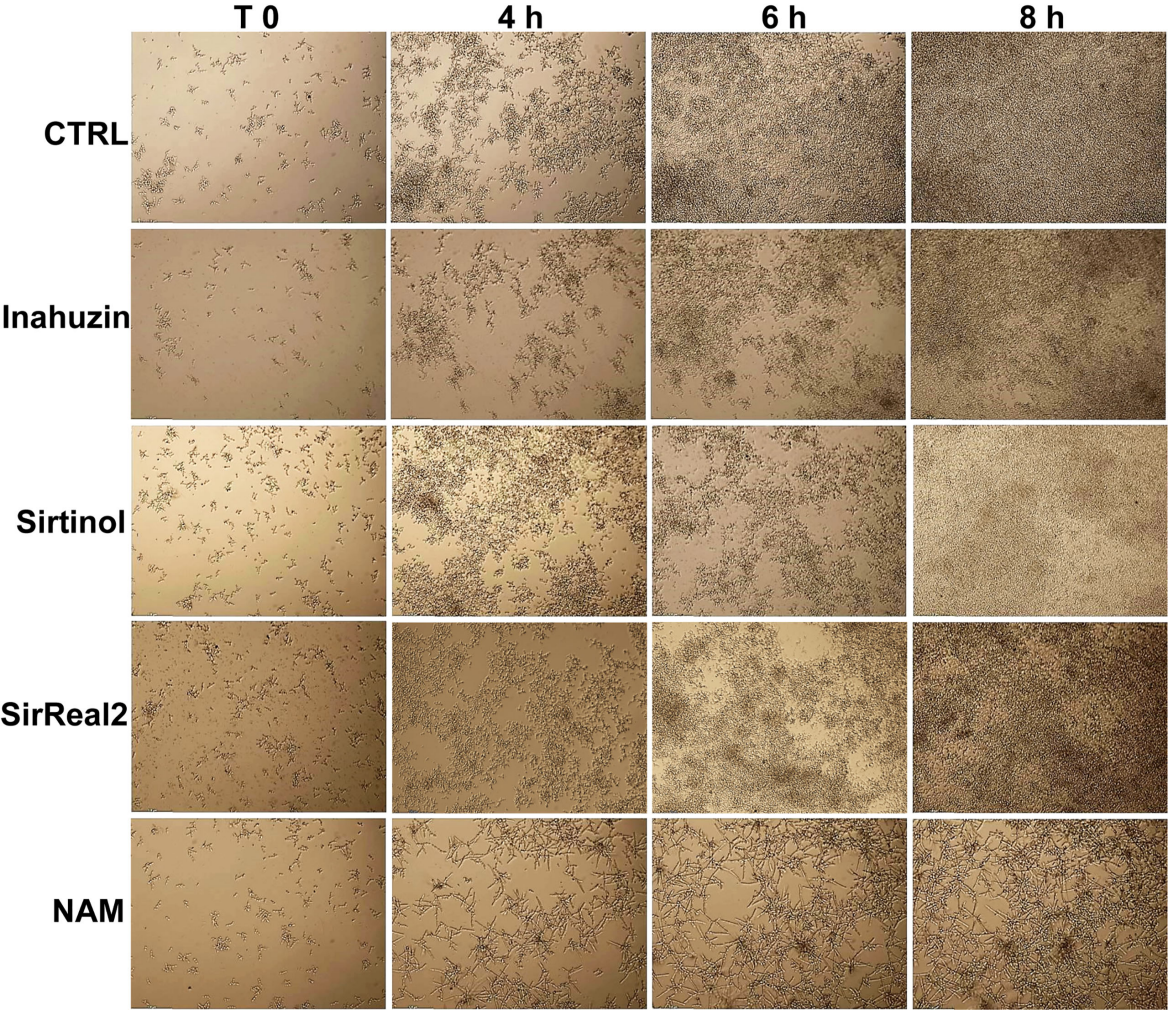
Morphological analysis of *C. albicans* in the presence of sirtuin inhibitors. *C. albicans* cells were treated with 50 µM Inauzhin, 10 µM Sirtinol, 50 µM SirReal2, or 10 mM NAM, and the morphology was imaged by Time-Lapse microscopy (10X magnification). Sirtinol, SirReal2, and Inauzhin do not inhibit Hst3p as they do not alter yeast morphology.

As a member of a NAD^+^-dependent histone deacetylases family, Hst3p is inhibited, in a non-specific and non-selective way, by NAM. In order to verify if commercially available sirtuin inhibitors affect Hst3p activity, we tested Sirtinol, SirReal2, and Inauhzin, which are specific SIRT1 and SIRT2 inhibitors. In detail, yeast cells from an overnight culture were inoculated in YPD containing 10 µM Sirtinol, 50 µM SirReal2, or 50 µM Inauhzin (the highest concentrations at which the inhibitors are fully soluble), or with 10 mM NAM, here used as control. Yeast growth was followed up to 24 h by Time Lapse imaging. No morphological alterations were observed with any of the inhibitors used, whereas cells treated with NAM formed hyphae with V-shaped branches as expected (**Figure 3**).

**Figure 4:**
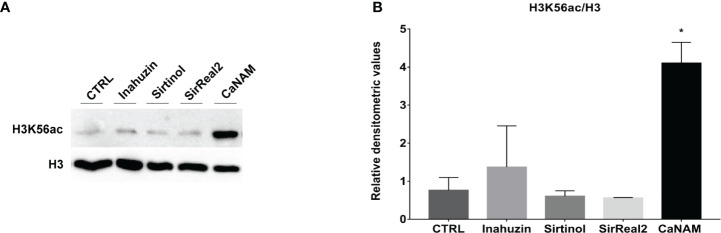
Sirtinol, SirReal2, and Inauzhin treatments do not result in H3K56ac accumulation. **(A)** *Candida* cells were treated with 50 µM Inauzhin, 10 µM Sirtinol, 50 µM SirReal2 and 10 mM NAM for 16 hours, and H3K56ac was analyzed by Western blotting; **(B)** Densitometric analysis of H3K56ac normalized to H3 from **(A)**. Values are mean ± standard error of three independent experiments *p<0.05 (t-test).

Moreover, Western blotting of histones extracted from overnight treatments confirmed that Sirtinol, SirReal2, and Inauhzin did not induce a significant accumulation of H3K56ac, whereas NAM inhibited Hst3p as showed by the higher level of acetylated H3K56 (**Figure 4**).

### Genome-wide analysis of H3K56ac

**Figure 5:**
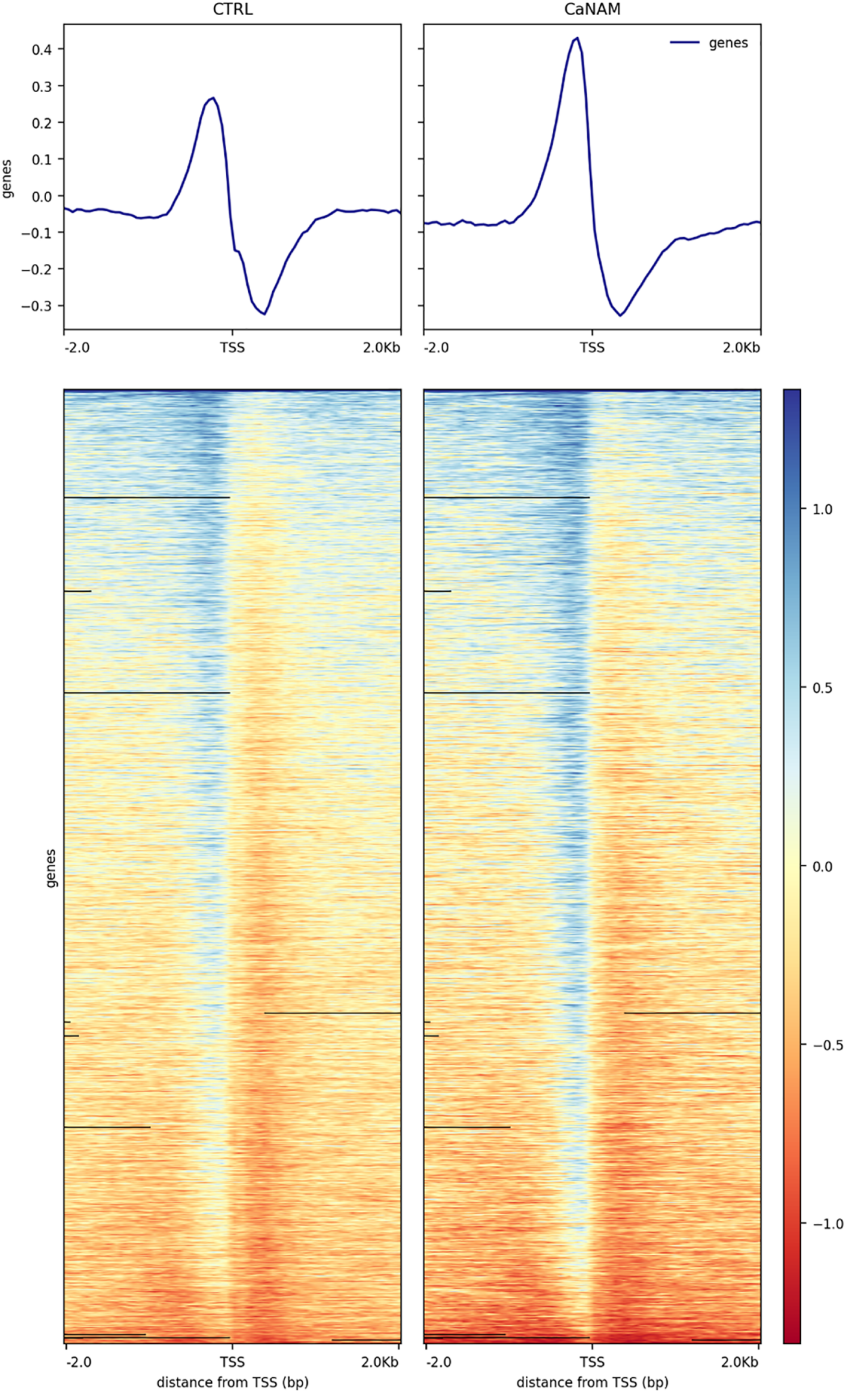
Profile heatmap around TSS of RefSeq genes. Representative heatmaps showing the scores associated with genomic regions around the TSS of genes (± 2 kb). The gradient blue-to-red color indicates a high-to-low log_2_ ratio of the number of reads between the IP and the respective Input in the corresponding region.

**Figure 6:**
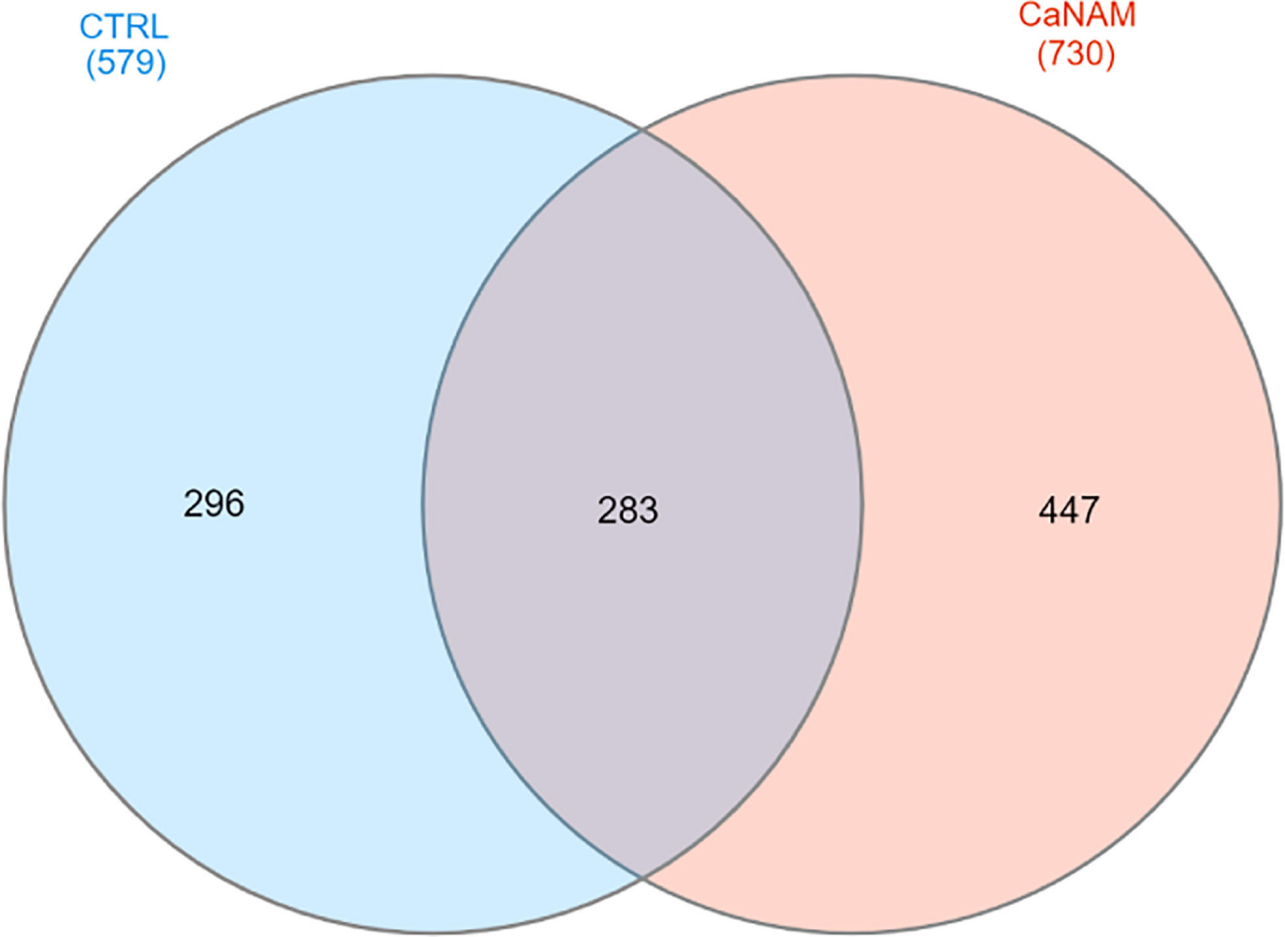
Venn diagram summarizing the number of ChIP enriched regions in CTRL and CaNAM. ChIP peaks annotations were plotted by InteractiVenn.

ChIP-seq is a powerful tool for the analysis and mapping of epigenetic marks. In order to identify the H3K56ac patterns across the *Candida albicans* genome, control and NAM-treated V-shaped cells were analyzed by ChIP-seq with anti-H3K56ac antibody after 28 h of incubation in YPD at 25°C. Plot profiles in **Figure 5** show that H3K56ac is mainly localized in genomic regions across the TSS of genes. We identified 671 and 843 ChIP-enriched regions in CTRL and CaNAM, respectively (**Supplementary Dataset 1**) (**Figure 6**). Since histone acetylation is mainly associated with open chromatin and consequent transcriptional activation, we focused on ChIP-enriched promoter regions (2 kb upstream TSS). Among them, 283 enriched regions are common to both experimental conditions and are mainly in the promoters of essential genes involved in metabolic process, transcriptional and translational control (i.e., transcription initiators, ribosomal proteins, translation elongator factors) (**Supplementary Figure S2**). Regions enriched for this histone modification only upon NAM treatment were 447, indicating that in those regions, H3K56 is likely deacetylated by Hst3p in control cells (**Figure 6**).

**Figure 7:**
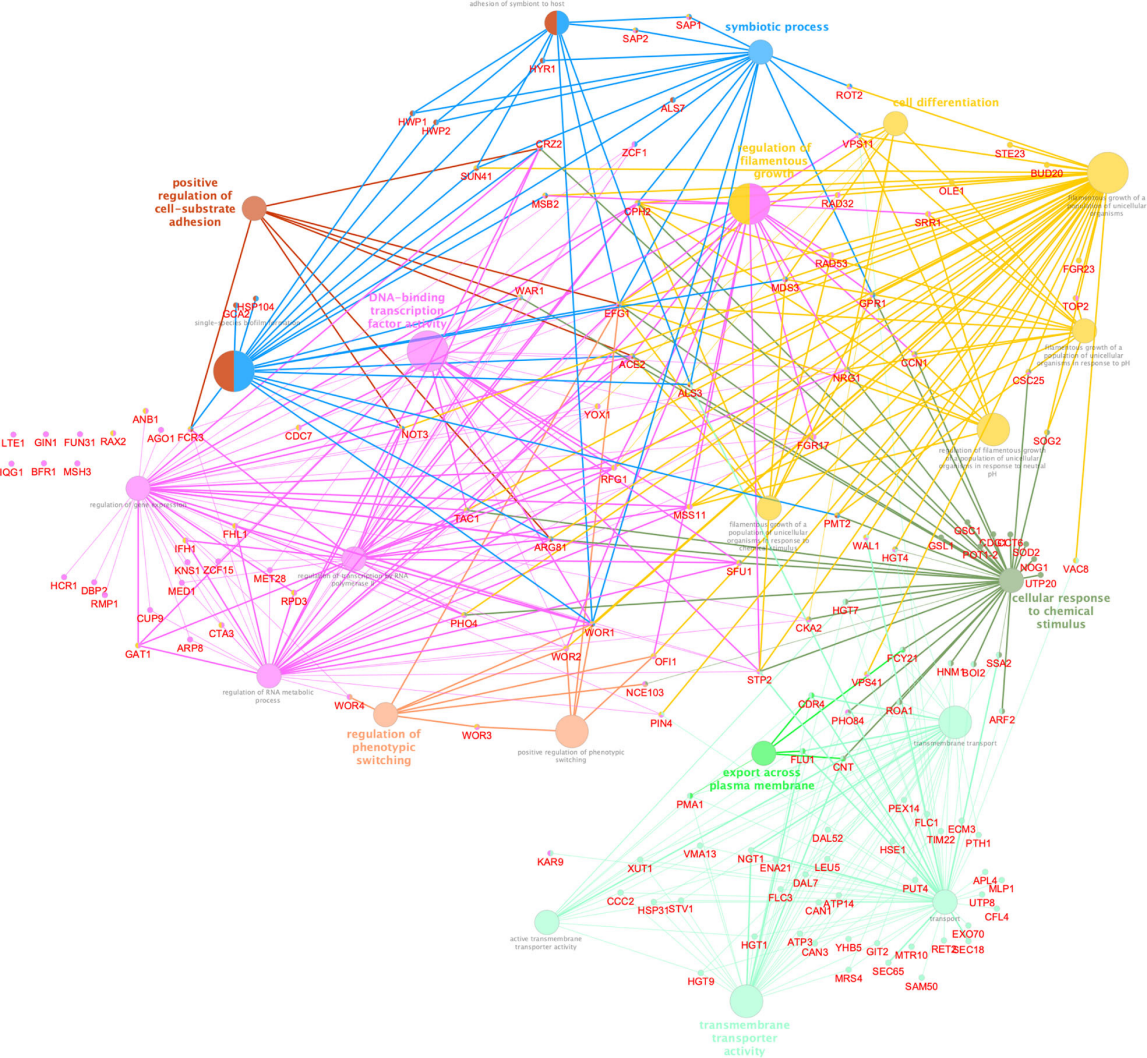
Cytoscape-based network analysis of 447 ChIP-enriched regions in CaNAM only. Biological process enrichment analysis using ClueGO and CluePedia Cytoscape plugins. The size of nodes indicated the degree of significance. The most significant term defined the group name (Bonferroni adjusted pValue ≤0.05).

To better understand the biological significance of differentially acetylated regions, we performed a functional analysis using ClueGo and CluePedia Cytoscape plugins, which integrates Gene Ontology (GO) terms to create a functionally organized GO term network, and we found significant enrichment for 8 GO biological processes (Bonferroni adjusted pValue ≤0.05). These differentially acetylated regions include promoters of genes involved in filamentation, phenotypic switching, and adhesion which would explain the abnormal V-shaped morphologies observed in CaNAM (**Figure 7**). Among them, we found several transcription factors involved in morphological processes: *OFI1*, involved in the regulation of white-opaque switching and filamentous growth (Du et al., 2015); *WAL1*, involved in polarized hyphal growth (Walther and Wendland, 2004); *EFG1*, a central transcriptional regulator of morphogenesis and biofilm (Glazier, 2022); *NRG1*, a transcription factor/repressor regulating hyphal gene and virulence (Braun et al., 2001); *ACE2*, which regulates morphogenesis, adherence, and virulence (Kelly et al., 2004); *WOR1*, the master regulator of white-opaque phenotypic switching (Huang et al., 2006), *WOR2*, *WOR3* and *WOR4* which, together with *WOR1*, *CZF1*, *EFG1*, and *AHR1*, form the transcriptional network that triggers the ability to switch between white and opaque cell types (Hernday et al., 2013; Lohse and Johnson, 2016). In addition, we found genes involved in adhesion and biofilm formation, such as *HWP1*, a hypha-specific cell surface protein required for biofilm formation *in vivo* (Nobile et al., 2006), and *HWP2*, a GPI-anchored protein required for biofilm formation, adhesion, filamentous growth (Hayek et al., 2010), the transcription factor *CRZ2*, required for pH-induced filamentation (Kullas et al., 2007), the adhesins *ALS3*, a fungal invasin that mimics host cell cadherins and induces endocytosis by binding to N-cadherin on endothelial cells and E-cadherin on oral epithelial cells (Phan et al., 2007).

### Transcriptome analysis

**Figure 8:**
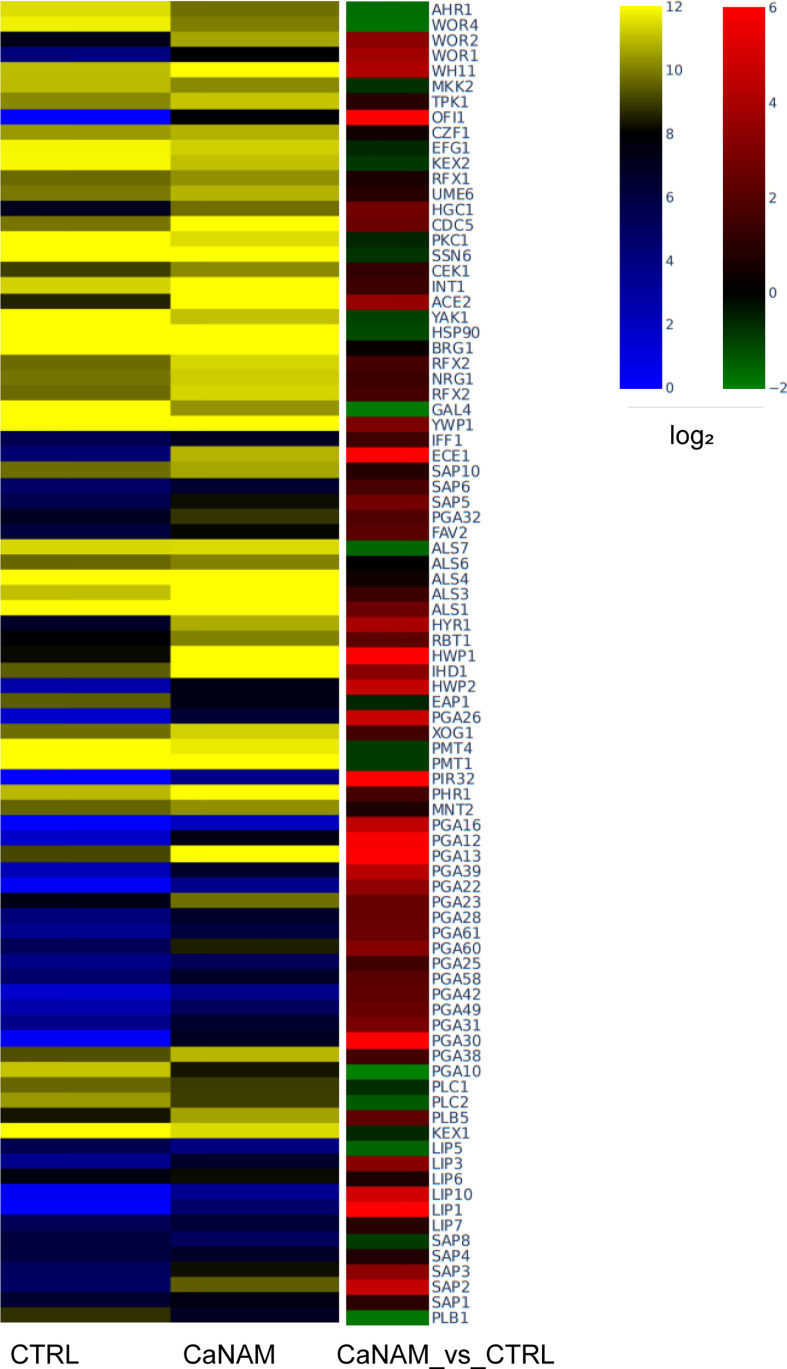
Dysregulated genes upon 10 mM NAM treatment. Heatmap showing log_2_ expression levels in RPKM (left) and log_2_ fold difference value (right) of selected upregulated genes involved in *C. albicans* virulence (Fold Change, |FC|≥1.5, FDR <0.05).

To integrate and strengthen ChIP-seq data and to better understand how H3K56 acetylation levels influence the *C. albicans* transcriptome and, consequently, the V-shaped hyphae development, we performed RNA sequencing of CaNAM *vs.* CTRL cells. We found 1330 upregulated (FC ≥ 1.5; FDR ≤ 0.05) and 1081 downregulated (FC ≤ -1.5; FDR ≤ 0.05) transcripts in CaNAM compared to CTRL cells (**Supplementary Figures S3 and 4**). Consistent with V-shaped morphology, Gene Ontology analysis revealed that the upregulated genes are mainly related to white-opaque, filamentation, cell wall organization, and adhesion (**Figure 8**).

Intersecting RNA sequencing data with the 447 ChIP-enriched regions of NAM-treated samples, we found 87 genes whose up-regulation directly correlates with H3K56 acetylation patterns associated with their promoters, including *OFI1*, *WOR1*, *WOR2*, *HWP1*, *NRG1*, *ACE2*, *CRZ2*, the secreted aspartyl proteinases *SAP1* and *SAP2*, aside from some GPI-anchored adhesin-like protein known to be involved in adhesion and virulence (**Supplementary Dataset 2**). Unexpectedly, a subset of 85 genes whose promoters displayed H3K56 acetylation only upon NAM inhibition showed an opposite trend, with higher acetylation but lower transcript abundance in CaNAM compared to CTRL, suggesting that H3K56 acetylation is not always followed by a higher transcription rate. This could be explained by the fact that in all eukaryotic organisms, a wide range of mechanisms contribute to transcription control (including mRNA stability control, Pol II termination, and Pol II attenuation) and is consistent with NET-seq analysis carried out by Topal and colleagues (Topal et al., 2019). They showed that, besides the leading role in enhancing transcription initiation, H3K56ac might transiently function as a transcriptional repressor by promoting nucleosome assembly in *S. cerevisiae*. Furthermore, our ChIP-seq analysis revealed that Hst3p also regulates H3K56ac across the promoters of genes encoding the RNA polymerase II mediator complex subunits MED1, the RNA polymerase II regulator EED1, besides other predicted ribonucleases or putative proteins with a predicted role in mRNA 3’-end processing, mRNA polyadenylation, and pre-mRNA cleavage.

These results suggest a possible role of H3K56ac in the modulation of a fine-tuning mechanism of transcriptional control.

In addition to the 87 genes whose expression seems directly related to H3K56ac associated with their promoters, our transcriptome analysis showed a subset of genes whose expression is regulated indirectly by such histone mark. For instance, our ChIP-seq revealed that Hst3p inhibition results in H3K56 acetylation associated with the promoter of *EFG1*, a key transcription regulator of several morphogenesis and virulence-related genes, whose transcription is not only negatively autoregulated by Efg1, but is also repressed by a series of positive feedback loops established by Wor1, Wor2, and Czf1 (Hernday et al., 2013). Moreover, *ECE1*, which encodes for the cytolytic peptide candidalysin, and *UME6*, the filament-specific transcriptional regulator, are up-regulated in RNA-seq upon NAM treatment and directly regulated by *EFG1* (Banerjee et al., 2008; Moyes et al., 2016).

Together our results reveal a complex mechanism behind the gene regulation mediated by H3K56ac, underlining the importance of such histone mark in controlling a wide range of biological processes in *C. albicans.*

## Discussion

Nosocomial infections caused by *Candida* spp. arise great concern due to the emergence of resistant fungal strains to the main antifungal drugs. In particular, *C. albicans* is the most prevalent species causing disease in both adult and pediatric populations, with a 30-day mortality rate approaching 40% in immunocompromised hosts (Koehler et al., 2019). The similarity between fungal and human cells limits the identification of specific targets that uniquely affect the microbial cell without damaging the host, further reducing the therapeutic options for treating fungal infections (Salazar et al., 2020). In addition, the intrinsic resistance to drugs such as azoles, echinocandins, or polyenes (Nami et al., 2019) of some pathogenic *Candida* species, brings the further need to develop antifungals with novel mechanisms for medical practice (Caruso et al., 2020).

In *C. albicans*, the most abundant post-translational modification is the H3K56 acetylation, regulated by the histone acetyltransferase Rtt109p and the histone deacetylase Hst3p. Histone acetylation/deacetylation balance plays a crucial role in *C. albicans* growth and virulence (Su et al., 2020). Interestingly, Hst3p displays sequence motifs not shared with human sirtuins (Wurtele et al., 2010); therefore, this histone deacetylase represents a promising therapeutic target for developing new antifungal agents. Several known HDAC inhibitors have already been explored as new potential antifungal therapy, but the selectivity of such molecules remains the main problem (Su et al., 2020). Indeed, we tested three currently available sirtuin inhibitors (Inauhzin, Sirtinol, and SirReal2), but none showed activity on Hst3p since they did not induce either morphological alterations in *Candida* or a significant accumulation of H3K56ac.

Therefore, we enrolled NAM as a non-specific sirtuin inhibitor to explore the effects of H3K56ac accumulation in *Candida albicans*. First, we examined the trend of H3K56 acetylation levels during the *Candida* yeast growth curve. In particular, by nano-ESI-LC-MS/MS, we observed that H3K56 acetylation reaches a maximum at the beginning of the logarithmic phase and then decreases, consistent with the observation that the H3K56ac marks newly synthesized histones, facilitating their deposition onto chromatin. On the contrary, when Hst3p is inhibited by NAM during yeast growth, we observed an accumulation of H3K56 acetylation over time, reaching a plateau at 24 h. Moreover, Hst3p inhibition results in abnormal phenotypes; in particular, when *Candida* is grown under hyphae-inducing conditions, there is a substantial filamentation reduction. In contrast, as previously observed by Wurtele and colleagues (Wurtele et al., 2010), when Hst3p is inhibited under yeast-promoting conditions, *Candida* forms an abnormal phenotype named V-shaped hyphae. However, we observed that the overall acetylation levels of H3K56 do not change significantly between yeasts, germ tubes, and hyphae, suggesting that this histone modification, weakening inter-nucleosomal interactions and leading to the chromatin relaxation for transcription (Wang and Hayes, 2008), is necessary for all morphogenetic phases of *Candida*.

Since the V-shaped hyphae is a peculiar morphology associated with increased levels of H3K56ac, we used these cells as a model to identify Hst3p targets. Although H3K56 acetylation is widespread in *Candida*, our ChIP-seq analysis revealed a strong enrichment across the TSS of genes related to *Candida* sp. pathogenicity, and morphology. Noteworthy, we identified 447 regions ChIP enriched only upon NAM treatment, including genes encoding for HSG, adhesin proteins, degradative enzymes, and white-opaque switching, such as *OFI1*, *ACE2*, *WOR1*, *WOR2*, *WOR3*, *WOR4*, *HWP1*, *HWP2, CRZ2*, *ALS3.* Moreover, our RNA-seq analysis showed a significant transcriptome dysregulation upon Hst3p inhibition, with 1330 up-regulated and 1081 down-regulated transcripts in 28 h CaNAM compared to CTRL. Gene Ontology analysis confirmed that, among the up-regulated transcripts, there are genes mainly related to filamentation, cell wall organization, and adhesion giving rise to the possibility that H3K56, through its acetylation status, regulates these processes.

Interestingly, we identified 87 genes whose transcriptional increase well correlates with the enrichment of H3K56 acetylation on their promoters, including some well-known regulators of phenotypic switching and virulence. For instance, the zinc-finger transcription factor *OFI1*, whose over-expression promotes filamentous growth in several culture conditions (Du et al., 2015) and *WAL1*, required for the organization of the cortical actin cytoskeleton and for the polarized hyphal growth (Walther and Wend land, 2004).

Moreover, our ChIP-seq revealed the presence of H3K56ac patterns in the promoter of *EFG1*, whose transcript abundance is lower in CaNAM compared to CTRL. This is consistent with previous studies showing that, overexpression of *EFG1* facilitates hyphal initiation but, shortly thereafter, leads to Efg1 dependent transcript down-regulation (Tebarth et al., 2003; Lassak et al., 2011). Furthermore, *EFG1* is repressed both directly and indirectly by Wor1 (Hernday et al., 2013) that is up-regulated upon NAM treatment. Efg1, acting *via* cAMP-PKA pathway, regulates the expression of several genes involved in the filamentation, such as *UME6*, a known regulator of hyphal extension, upregulated in the V-shaped conditions (Banerjee et al., 2008). These results might explain the abnormal phenotype, namely V-shaped hyphae, observed in *C. albicans* cells exposed to NAM in yeast promoting conditions.

We are aware that the use of a non-specific inhibitor may not be the best way to proceed, but several indications suggest that the effects of NAM reported in this study are exerted mainly through inhibition of Hst3p activity. Firstly, in *C. albicans*, the deacetylation of H3K56 occurs *via* Hst3p, thus the accumulation of H3K56ac following NAM treatment is likely due to the inhibition of Hst3p. In addition, in our experimental conditions, we used a concentration of NAM that does not affect *Candida* growth, but induces the formation of V-shaped hyphae, the same phenotype observed by Wurtele and coworkers in the heterozygous deletion mutant of *HST3*. Moreover, although in *C. albicans*, besides Hst3p there are other sirtuins, such as Sir2p, Hst1p, and Hst2p, Zhao and Rusche (2021) demonstrated that true hypha formation was significantly reduced only by the deletion of *SIR2* but not of *HST1* or *HST2*. Moreover, the expression of hypha-specific genes *HWP1*, *ALS3*, and *ECE1* was significantly reduced in a *sir2* single mutant compared to the wild type. In contrast, in our experimental conditions, all three of these genes are up-regulated, suggesting that the inhibition of Hst3p obtained with NAM predominates over that eventually exerted on Sir2p.

Overall, our study represents the first genome-wide H3K56 acetylation analysis in *C. albicans* and provides the first map of H3K56ac patterns across the *C. albicans* genome, representing a rich resource for future studies. Nevertheless, the evidence that H3K56ac directly regulates the expression of several virulence-related genes points out the relevance of such epigenetic modification in regulating *C. albicans* virulence, confirming that Hst3p is an appealing target for the development of new potential antifungal drugs.

## Data availability statement

The datasets presented in this study can be found in online repositories. The name of the repository and accession number(s) can be found below: ArrayExpress, accession E-MTAB-12193; E-MTAB-12167.

## Author contributions

MC: data curation, investigation, methodology, visualization, bioinformatic analysis, writing—original draft preparation, and writing—review and editing; DE: data curation, investigation, methodology, visualization, and writing—review and editing; MP: investigation and methodology; AMP: investigation and methodology; MM: methodology and writing—review and editing; CC: methodology; GG: methodology and bioinformatic analysis; FR: methodology and bioinformatic analysis; PT: methodology and writing—review and editing, AnP: data curation and investigation; AT: conceptualization, data curation, investigation, validation, writing—review and editing, and funding acquisition; AmP: conceptualization, data curation, investigation, validation, visualization, writing—original draft, writing—review and editing, and funding acquisition. All authors contributed to the article and approved the submitted version.

## Funding

This work was supported by University of Salerno intramural funds FARB.

## Conflict of interest

The authors declare that the research was conducted in the absence of any commercial or financial relationships that could be construed as a potential conflict of interest.

## Publisher’s note

All claims expressed in this article are solely those of the authors and do not necessarily represent those of their affiliated organizations, or those of the publisher, the editors and the reviewers. Any product that may be evaluated in this article, or claim that may be made by its manufacturer, is not guaranteed or endorsed by the publisher.
