# Supplementary material for: Effects of Hst3p inhibition in Candida albicans: a genome-wide H3K56 acetylation analysis
Source: Front Cell Infect Microbiol. 2022 Oct 27;12:1031814. doi: 10.3389/fcimb.2022.1031814 (PMC9647175; doi:10.3389/fcimb.2022.1031814)
Supplement: Supplementary file 1 [file DataSheet_1.pdf]

Supplementary figures

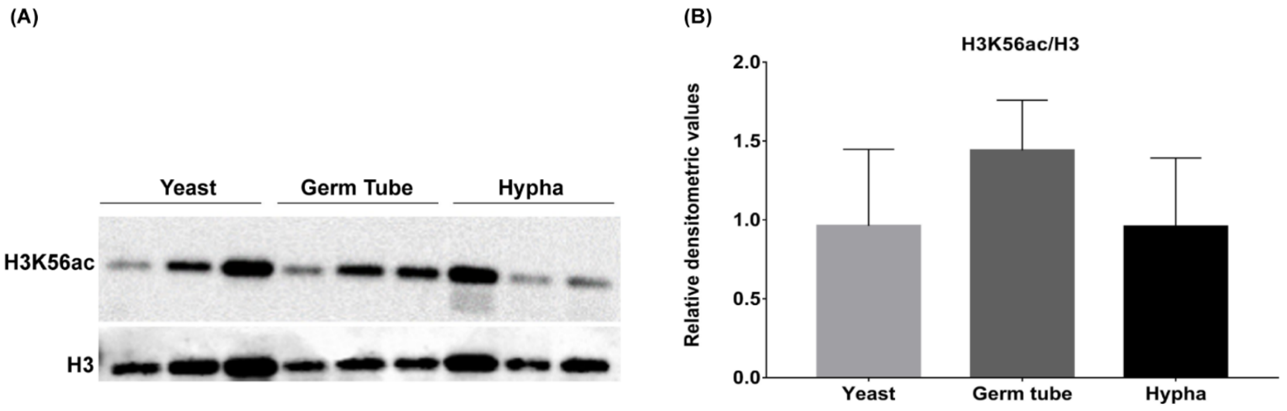

Supplementary Figure 1: (A) Western blotting showing H3K56ac levels yeast, germ tube and hypha. (B) Densitometric analysis of H3K56ac normalized to H3 from three independent experiments. Values are mean ±standard error of three independent experiments ***p<0.001 (t-test).

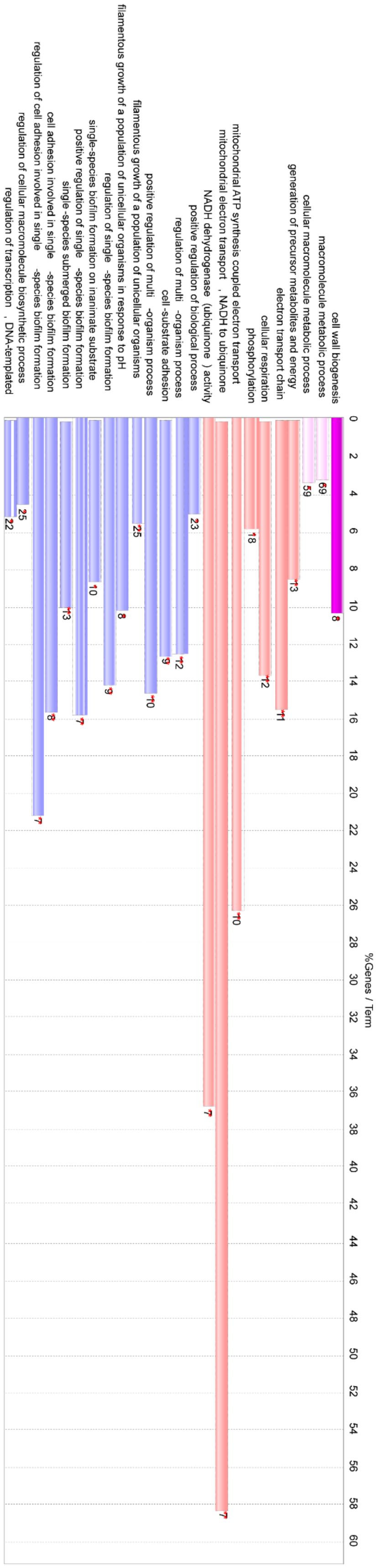

Suppl. Figure 2: Gene ontology analysis of 283 regions enriched in both CTRL and CaNAM. Biological process enrichment analysis using ClueGO Cytoscape plugins. The bars represent the number of genes associated with the terms.

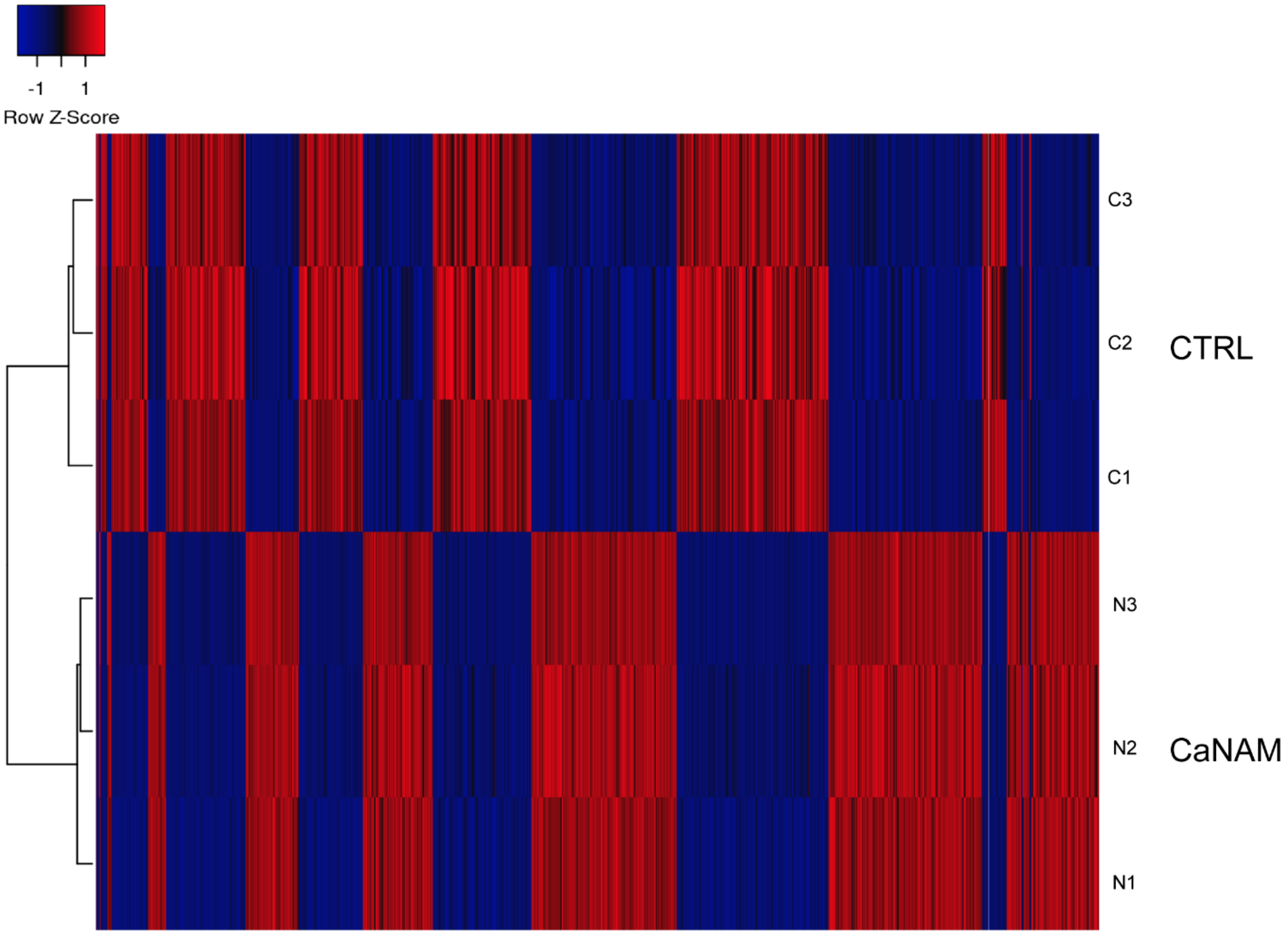

Supplementary Figure 3: Dysregulated genes upon 10 mM NAM treatment. Heatmap showing the expression levels in log2 RPKM of differentially expressed genes upon NAM treatment (C-1;2;3= control replicates; N- 1;2;3= NAM treated replicates) (FDR ≤0.05).

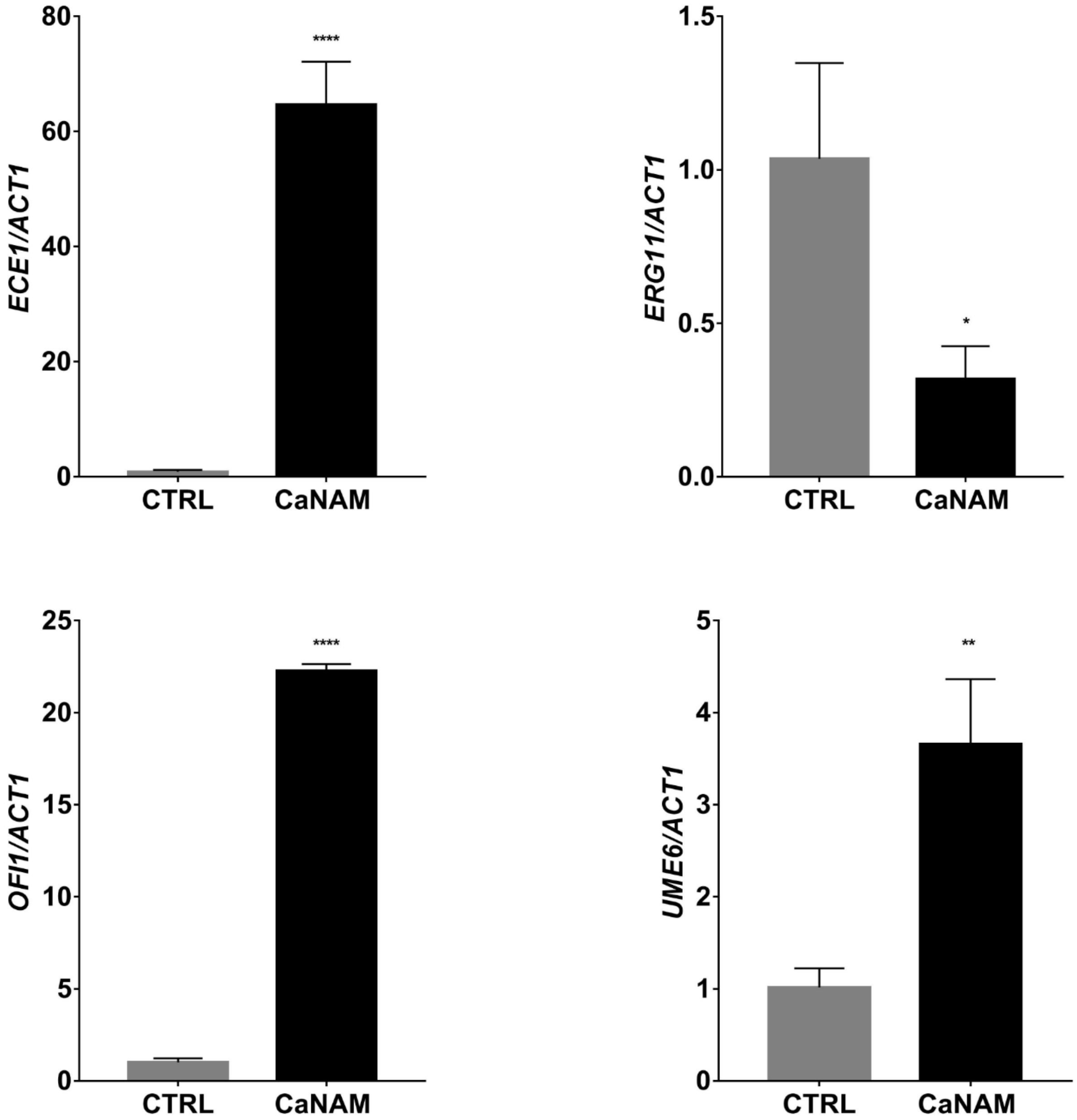

Suppl. Figure 4: validation of RNA-seq result. RT-q-PCR analysis of some representative genes resulted dysregulated in RNA-seq. *p<0.05; **p<0.01; ***p<0.001 (t-test).

Supplementary methods

RT-q-PCR

1 µg of each RNA was retrotranscribed by M-MLV Reverse Transcriptase (GeneSpin S.r.l, #STS-MRT, Italy). The Real-time PCR was performed using the Light Cycler 480 II instrument (Roche, Basel, Switzerland). Suitable dilutions of cDNA were used for each gene in a 12 µL reaction using Luna Universal qPCR Master Mix (New Englands BioLabs, #M3003, USA). The primers sequences are reported in Supplementary Table 1. Results from 3 independent experiments in technical duplicates were analyzed using the Delta-Delta CT method and ACT1 as a reference gene.

Supplemetary Table 1: List of primers used for RT-q-PCR.

GENE	FORWARD	REVERSE
ACT1	AAGAATTGATTTGGCTGGTAGAGA	TGGCAGAAGATTGAGAAGAAGTTT
ERG11	TGACCGTTCATTTGCTCAAC	GCAGCATCACGTCTCCAATA
OFI1	CAAGGCTGGACCCACAGACA	ATTGCTGTGACTGGGCTGGT
ECE1	CTAATGCCGTCGTCAGATTG	AACATCTGGAACGCCATCTC
UME6	CCCAGCACTGCTACTGGATCT	GGTTGGGATTGTGCTTGTTGT
